# Chemotherapy Toxicity in Older Adults Optimized by Geriatric Assessment and Intervention: A Non-Comparative Analysis

**DOI:** 10.3390/curroncol29090484

**Published:** 2022-08-26

**Authors:** Munzir Hamid, Michelle Hannan, Nay Myo Oo, Paula Lynch, Darren J. Walsh, Tara Matthews, Stephen Madden, Miriam O’Connor, Paula Calvert, Anne M. Horgan

**Affiliations:** 1 Oncology Department, University Hospital Waterford, X91 ER8E Waterford, Ireland; 2 Data Science Centre, RCSI University of Medicine and Health Sciences, D02 YN77 Dublin, Ireland

**Keywords:** chemotherapy, toxicity, geriatric assessment, healthcare utilization

## Abstract

The Comprehensive Geriatric Assessment (CGA) is recommended to guide treatment choices in older patients with cancer. Patients ≥ 70 years referred to our oncology service with a new cancer diagnosis are screened using the G-8. Patients with a score of ≤14 are eligible to attend the Geriatric Oncology and Liaison (GOAL) Clinic in our institution, with referral based on physician discretion. Referred patients undergo multidimensional assessments at baseline. CGA domains assessed include mobility, nutritional, cognitive, and psychological status. Chemotherapy toxicity risk is estimated using the Cancer Aging and Research Group (CARG) calculator. We undertook a retrospective analysis of patients attending the GOAL clinic over a 30-month period to April 2021. The objective was to determine rates of treatment dose modifications, delays, discontinuation, and unscheduled hospitalizations as surrogates for cytotoxic therapy toxicity in these patients. These data were collected retrospectively. Ninety-four patients received chemotherapy; the median age was 76 (70–87) and 45 were female (48%). Seventy-five (80%) had an ECOG PS of 0–1. Seventy-two (77%) had gastrointestinal cancer, and most had stage III (47%) or IV (40%) disease. Chemotherapy with curative intent was received by 51% (*n* = 48) and 51% received monotherapy. From the CGA, the median Timed Up and Go was 11 s (7.79–31.6), and 90% reported no falls in the prior 6 months. The median BMI was 26.93 (15.43–39.25), with 70% at risk or frankly malnourished by the Mini Nutritional Assessment. Twenty-seven (29%) patients had impaired cognitive function. Forty-three (46%) had a high risk of toxicity based on the baseline CARG toxicity calculator. Twenty-six (28%) required dose reduction, 55% (*n* = 52) required a dose delay, and 36% (*n* = 34) had a hospitalization due to toxicity. Thirty-nine patients (42%) discontinued treatment due to toxicity. Despite intensive assessment, clinical optimization and personalized treatment decisions, older adults with cancer remain at high risk of chemotherapy toxicity.

## 1. Introduction

Cancer is a disease of older adults, with 48% of people diagnosed over the age of 70, with this expected to rise to 58% by the year 2035 [1]. Oncologists are interested in valid and reliable tools to assess older patients undergoing cancer therapy in an effort to reduce treatment toxicities, decrease unnecessary delays, and better guide dose modifications [2,3,4,5]. Concerns regarding treatment tolerance drive the use of treatment modifications based on chronological age [6,7,8] rather than functional status [9,10]. Toxicity is affected not only by changes in body organ function [11], but also by other factors, such as nutritional [12], psychological factors [13,14], and polypharmacy [15].

The American Society of Clinical Oncology (ASCO), The International Society of Geriatric Oncology (SIOG), the National Cancer Control Network (NCCN), and others, recommend a Comprehensive Geriatric Assessment (CGA) for older patients with cancer [16,17,18,19]. The CGA is a multidimensional assessment of the functional status, comorbidities, mental health, social support, polypharmacy, and nutritional status of older adults [20,21]. Several prospective cohort studies have shown the benefit of the CGA in the assessment of older patients undergoing cancer therapy, leading to a better prediction of toxicity risk, and thus guiding treatment choices [22,23,24,25]. Data show that CGA contributes to cancer therapy decision-making in 20–50% of cases [3,16,25], with functional status, nutritional status, and comorbidities having the highest impact on decision-making [26,27,28]; functional impairment and older age increases the likelihood of dose modification of cytotoxic drugs [29]. Interventions driven by CGA have been shown in randomized controlled trials to increase the probability of completing scheduled chemotherapy, decreasing rates of dose reductions [30], and leading to significant reductions in serious cytotoxic treatment side-effects [31,32]. Integrating assessment with geriatrician leadership has also proven beneficial in improving health-related quality of life and decreasing both hospital admissions and treatment discontinuation in older adults receiving systemic therapy [33].

The Geriatric Oncology and Liaison (GOAL) Clinic in our centre is a service developed specifically for patients aged 70 and older with a solid cancer diagnosis, and it is the first dedicated geriatric oncology clinic at a major Irish cancer centre. New patients referred to medical oncology are screened using the G-8 screening tool [34]. Those scoring ≤ 14 are deemed suitable for the full CGA, which incorporates assessments of function, mobility, nutrition, psychosocial wellbeing, and cognition. All patients undergo a pharmacist-directed drug reconciliation and quantification of toxicity risk using validated tools. Outcomes generate multidisciplinary input by specialists in geriatric medicine, psychiatry of old age services, occupational therapy, physiotherapy, dietetic, and social work services where indicated. 

We sought to determine the rates of treatment dose modifications, delays, discontinuation, and unscheduled hospitalizations as surrogates for cytotoxic therapy toxicity in patients attending the GOAL clinic. Furthermore, we aimed to determine if the CGA variables not incorporated in established prediction tools (CRASH/CARG) [35,36] could predict treatment toxicity.

## 2. Materials and Methods

### 2.1. GOAL Clinic

The GOAL clinic was developed in University Hospital Waterford, South East Cancer Centre. This is a teaching hospital, and one of the eight designated cancer centres in Ireland, serving a population of c500,000. Referral to the GOAL clinic is based on age, G-8 scores, and physician discretion. G-8 screening is undertaken by an Advanced Nurse Practitioner (ANP). This was initially restricted to patients with gastrointestinal malignancies. The GOAL clinic has evolved, and the G-8 is now completed on all patients ≥ 70 years referred to the medical oncology department prior to the first review. The G-8 results are relayed back to the assigned medical oncologist with a suggestion to refer to the GOAL clinic when G-8 ≤ 14. The ultimate decision to refer is with the treating oncologist, with a 70% referral rate noted. The CGAs completed are outlined below. These are undertaken by the ANP on the day of the first clinical consultation. The results are available to the oncology team when first assessing the patient and assist in directing treatment choice and baseline dosing. The CARG toxicity risk can aid in the dosing decision but does not dictate it—the ultimate decision relies on clinical discretion. All data are uploaded to an excel data sheet post-review. Interventions are suggested based on any deficits identified and appropriate referrals are initiated by the ANP. (See Supplementary Table S1). Fast track access to the Waterford Integrated Care for Older Patients (WICOP) has been established to facilitate rapid review of patients where indicated. 

### 2.2. Overall Design

We undertook a retrospective analysis of data collected on all patients seen in the GOAL clinic at our institution over a 30-month period to April 2021. We only included patients who received cytotoxic treatment. Patients receiving hormonal therapy, immunotherapy, surgery, radiation therapy, or best supportive care alone were excluded. 

### 2.3. Data Collection

Data collected were baseline demographics (age and gender), cancer type and disease stage, treatment intent (curative or palliative), and chemotherapy regimen (monotherapy or polytherapy). The CGAs included the Timed Up and Go (TUG) [37], history of falls in the last 6 months (yes or no), and number of concomitant medications. Nutrition was assessed using the mini nutritional assessment (MNA) [38,39] and scores were interpreted as malnutrition (0–7), at risk of malnutrition (8–11), and normal (12–14). Body mass index was recorded. Depression was screened for using the 15-item Geriatric Depression Scale (GDS-15) [40], with scores ≤ 5 considered normal and scores of >5 suggested depression. We collected scores of Katz Activities of Daily Living (ADL) with scores between 0–6 (0 very dependent and 6 independent) [41], Lawton Instrumental ADL, a summary score ranges from 0 (low function, dependent) to 9 (high function, independent), and Eastern Cooperative Oncology Group (ECOG) Performance Status [42]. Comorbidities were collected using the Charlson Comorbidity Index [43] and quantified as mild (1–2); moderate (3–4); and severe (≥5). Cognitive assessment was performed using the Mini-cog test [33] or 6CIT [44]. If impairments were noted, then patients proceeded to a Montreal Cognitive Assessment (MOCA) [45]. Results were divided into cognitive impairment or no impairment. The results of chemotherapy toxicity risk, estimated using the Cancer Aging and Research Group (CARG) calculator [34], were categorized into low (0–5), intermediate (6–9), and high (≥10) risk. The rates of baseline chemotherapy dose reduction were collected. This was recorded in the GOAL database. We identified subsequent dose reductions (defined as any reduction from baseline dose), dose delays, treatment discontinuations, or unscheduled hospitalizations as surrogates of treatment toxicity. The chemotherapy pharmacy compounding unit provided data on dose delays, dose reductions during treatment, and treatment discontinuation. Reasons for discontinuation and hospitalizations were collected from retrospective review of patient records. We opted to use these outcomes as opposed to the NCI-CTCAE Grade 3–5 toxicity to ensure accuracy of reporting, where all grade 3 toxicity not requiring hospitalization may not be captured retrospectively.

### 2.4. Data Analysis

The mean, standard deviation, and range for continuous variables and the frequencies for categorical variables were calculated as part of a descriptive analysis using Microsoft Excel. Tests for the statistical significance of single variables were performed using the Student’s *t*-test, while two-variable correlations were assessed pairwise using the Fisher’s Exact Test.

We examined six potential pre- and post-treatment interactions. These were predetermined prior to analysis. Using Fisher’s Exact Tests with either 2 × 2 or 3 × 2 contingency tables, we sought to examine whether either the CARG risk score for each patient at baseline/pre-modification of treatment (low, medium, or high) or the presence/absence of cognitive impairment could be related to one of three post-treatment outcomes: dose reduction due to toxicity, discontinuation due to toxicity, or hospitalization due to toxicity. A *p* value of 0.05 was considered significant.

## 3. Results

Data on 94 patients aged ≥70 who received cytotoxic therapy were analysed. Patient demographics are summarized in Table 1. About half were male, 76% (*n* = 71) had an ECOG PS of 1 and 77% (*n* = 72) had a diagnosis of gastrointestinal cancer. Thirty eight percent (*n* = 38) had metastatic disease and forty nine percent (*n* = 46) were treated with polychemotherapy.

### 3.1. Comprehensive Geriatric Assessment

We gathered data on 11 variables for the CGA (Table 2). The median TUG score was 11 s (range 2.79–31.6). Only 9 of the 94 study participants (9%) had a fall within the last 6 months. Twelve patients (13%) scored > 5 on the geriatric depression scale, and more than half of patients (62%) scored ≥ 3 on the Charlson Comorbidity Index.

Nearly one-third of patients (29%) displayed cognitive impairment. Median BMI was 26.93 (15.43–39.25), with 70% at risk or frankly malnourished by the MNA. Using the CARG toxicity risk calculator, 47% (*n* = 44) were deemed at medium risk and 46% (*n* = 43) at high risk of toxicity.

### 3.2. Treatment Outcomes

The four toxicity-dependent outcomes are shown in Table 3. About one-third to one-half of all patients experienced toxicity severe enough to warrant some significant alteration in their treatment plans. A delay in the administration of subsequent doses was the most common outcome, with 55% (*n* = 52) of all patients requiring this modification. A total of 42%(*n* = 39) required a discontinuation of their treatment due to toxicity, and 36% (*n* = 34) required hospitalization with toxicity. Data on the prevalence of geriatric deficits in different subgroups of patients who experienced toxicities are shown in Supplementary Table S2.

### 3.3. Statistical Analysis of Toxicity Correlations

Six potential pre- and post-treatment interactions are outlined in Table 4. None of the associations exhibited a significant relationship. 

## 4. Discussion

Our data show the vulnerability of older patients with cancer to the toxic effects of chemotherapy and thus the importance of a multidisciplinary and tailored approach to each patient. Innumerable drug studies report the comparable benefit of systemic anticancer therapy in older and younger adults [46,47]. However, these studies nearly universally report the higher risk of toxicity in the older cohort. Thus, optimizing treatment decisions and generating bespoke treatment plans, incorporating the cancer diagnosis and stage, as well as the physiological and pathological changes in the older adult is essential. While unplanned hospitalization and dose modifications is an accepted component of cancer care, the rates in our group remain substantial, though comparable to larger, randomized studies recently reported [31,32].

The GAIN study [31] enrolled 613 patients aged ≥65 with a solid malignancy who were due to start a new chemotherapy regimen and had completed a CGA. Patients were randomized (2:1) to either the intervention or standard care arm. The primary outcome was the incidence of grade 3 or higher chemotherapy-related toxic effects. Secondary outcomes included emergency department visits, unplanned hospitalizations, chemotherapy dose modification, and early discontinuation. In this study, 27% had an emergency department visit and 22% had an unplanned hospitalization in the intervention arm. Fifty-four percent discontinued chemotherapy early and fifty-four percent required chemotherapy dose modifications (reductions and delays). In contrast to our service, a full geriatrics-trained multidisciplinary team was involved in reviewing the baseline assessments, in recommending appropriate interventions, and initiating referrals in the GAIN study.

The GAP70+ study [32] was a cluster-randomized trial, enrolling patients ≥ 70 years with incurable solid tumours or lymphoma. In this study, patients were required to have at least one impaired geriatric assessment domain. Patients were randomized to the intervention arm, where oncologists received a tailored geriatric assessment summary and management recommendations, or to the usual care arm, where no geriatric assessment or summary was provided to oncologists. We utilize a similar approach, however, in the GOAL clinic the referrals are initiated by the geriatric oncology ANP and are not solely at the discretion of the primary oncologist. The primary outcome in the GAP70+ study was the rate of grade 3–5 toxicities. In this study, a higher proportion of patients in the intervention arm received treatment at a reduced dose intensity than standard in cycle one (49%), and 43% required dose reductions because of toxic effects over three months in the intervention arm. 

While both the GAP and GAIN studies have proven the benefit of geriatric assessment and intervention in reducing significant toxic effects from cancer treatment, the rates of hospitalization and treatment modifications remain high in both studies. Our data showed similar findings, despite the use of the CGA, pre-treatment modifications in 26% based on the CARG toxicity calculator, and targeted interventions of any deficits identified on the baseline assessment. However, the rates would be significantly higher outside the setting of a geriatric-focused clinical service. INTEGERATE was a prospective, randomized, parallel group study in patients aged >70 years with cancer planned for chemotherapy, targeted therapy, or immunotherapy [33]. Patients were randomly assigned to receive either geriatrician-led comprehensive geriatric assessment or management integrated with usual care (integrated oncogeriatric care) or usual care alone. While the primary outcome was health-related quality of life measured by the Elderly Functional Index (ELFI) score, a major secondary outcome included healthcare utilization. In this study, there were 39% less emergency presentations, 41% less unplanned hospital admissions, and 24% less unplanned hospital overnight bed days in the integrated oncogeriatric care arm.

Other studies have reported correlations between the CARG risk score and toxicities in geriatric oncology patients [48,49,50]. Within our data set, there was no clear CARG risk status or cognitive impairment status that would accurately predict the toxicity-driven negative outcome of a chemotherapy treatment. We modified our initial treatment decisions based on the baseline CARG risk. Thus we can infer benefit from this approach as those at high risk at baseline were at no greater risk than medium or low risk after treatment modification. 

Our results came despite a seemingly healthy population at baseline. The median TUG score was 11 s; a score greater than 13.5 s is the convention to identify those at increased risk of falls in the community [37]. Only 9 of the 94 study participants (9%) had a fall within the last 6 months, while the literature reports that about 20–30% of elderly patients with cancer will fall within a year [51]. In addition, 80% had a performance status of 0 or 1. However, it is acknowledged that performance status is not a good marker in older adults [24,52]. On the other hand, the data identified vulnerabilities in terms of nutrition with high rates of malnutrition in at-risk groups and polypharmacy. Cognitive impairment was noted in 29% of patients, which is significantly higher than seen in the GAIN study (6.7%) and cognitive impairment reported in 40% of the GAP70+ study population. Two-thirds of patients had a significant co-morbidity burden, comparable to randomized studies. Consequently, the population was putatively at some risk for chemotherapy toxicity despite seemingly being healthy outside their oncological status.

The current study has some limitations, including its retrospective nature, relatively small sample size, no comparator population available, and we included predominantly a population with GI malignancies. Nonetheless, it is the only toxicity data from an Irish population of older patients seen in a dedicated geriatric-oncology service.

Our data confirm the significant risk of toxicity from cytotoxic therapy in an older population. Randomized data confirms the benefit of geriatric assessments and interventions to decrease toxicity risks. It is important to develop, support, and resource geriatric-oncology services to allow ongoing, multidisciplinary input throughout the course of treatment in an effort to proactively manage and modify treatments, decrease healthcare utilization, decrease treatment risks, and improve quality of life for our older patients with cancer.

## Figures and Tables

**Table 1:** Patient Demographics.

Variable (*n* = 94)	*n*	%
Age (years)		
Mean	76.49	—
Median (range)	76 (70–87)	—
Gender		
Male	45	48
Female	49	52
ECOG PS		
0	4	4
I	71	76
II	16	17
III	2	2
IV	1	1
Cancer Type		
Gastrointestinal	72	77
Breast	8	9
Genitourinary	5	5
Gynaecological	2	2
Lung	7	7
Cancer Stage		
I	1	1
II	11	12
III	44	47
IV	38	38
Treatment Intent		
Curative	48	51
Palliative	46	49
Treatment Regimen		
Monotherapy	48	51
Polytherapy	46	49

Eastern Cooperative Oncology Group Performance Status.

**Table 2:** Geriatric Assessment Variables.

Variable	*n*	%
Timed Up and Go		
Mean	11.99 s	—
Median (range)	11 (7.79–31.6) s	—
Falls in the last 6 months		
Yes	8	9
No	85	90
N/A	1	1
Concomitant meds		
Mean	6.32	—
Median (range)	6 (0–19)	—
Mini Nutritional Assessment (MNA)		
0–7	28	30
8–11	51	54
12–14	15	16
Body mass index		
Mean	27.02	—
Median (range)	26.93 (15.43–39.25)	—
Geriatric depression scale		
≤5	79	84
>5	12	13
N/A	3	3
Katz ADLs		
Mean	5.74	—
Median (range)	6 (3–6)	—
^Lawton IADLs^		
^ ^^Mean^	7.44	—
^ ^^Median (range)^	8 (3–8)	—
Charlson Comorbidity Index		
0	0	0
1–2	32	34
3–4	20	21
≥5	42	45
* Cognitive impairment		
Yes	27	29
No	61	65
N/A	6	6
CARG toxicity risk		
Low	4	4
Medium	44	47
High	43	46
N/A	3	3

N/A = not available; Activities of Daily Living; Instrumental Activities of Daily Living; Cancer and Aging Research Group. * Cognitive impairment defined as MOCA < 26.

**Table 3:** Treatment Outcomes.

Outcome	*n*	%
Baseline dose reduction		
Yes	24	26
No	67	71
N/A	3	3
Subsequent dose reduction		
Yes	26	28
No	60	64
N/A	8	8
Dose delay		
Yes	52	55
No	36	39
N/A	6	6
Discontinuation due to toxicity		
Yes	39	42
No	51	54
N/A	4	4
Hospitalization due to toxicity		
Yes	34	36
No	56	60
N/A	4	4

**Table 4:** Fisher’s Exact Tests for interdependence of pre- and post-treatment parameters.

Post-Treatment Status	Pre-Treatment Status	*p* Value
Dose reduction (Y/N)	CARG risk (Low/Med/High)	0.712 ^1^
Toxicity (Y/N)	CARG risk (Low/Med/High)	0.367 ^1^
Hospitalization (Y/N)	CARG risk (Low/Med/High)	0.509 ^1^
Dose reduction (Y/N)	Cognitive impairment (Y/N)	0.340 ^2^
Toxicity (Y/N)	Cognitive impairment (Y/N)	0.347 ^2^
Hospitalization (Y/N)	Cognitive impairment (Y/N)	1 ^2^

^1^ A 3 × 2 contingency table was used. ^2^ A 2 × 2 contingency table was used. Y = yes, N = No; CARG = Cancer and Aging Research Group.

## Data Availability

The data presented in this study are available on request from the corresponding author for scientific purposes.

## Supplementary Materials

The following supporting information can be downloaded at: https://www.mdpi.com/article/10.3390/curroncol29090484/s1, Table S1: Geriatric Assessments and interventions; Table S2: Toxicity subgroups and CGA deficits.
