# Supplementary material for: Chemotherapy Toxicity in Older Adults Optimized by Geriatric Assessment and Intervention: A Non-Comparative Analysis
Source: Curr Oncol. 2022 Aug 26;29(9):6167–76. doi: 10.3390/curroncol29090484 (PMC9498117; doi:10.3390/curroncol29090484)
Supplement: Supplementary file 1 [file curroncol-29-00484-s001.zip › curroncol-1779676-supplementary.pdf]

Supplementary Materials for Chemotherapy Toxicity in Older Adults Optimized by Geriatric Assessment and Intervention: A Non-Comparative Analysis

Supplementary Table S1: Geriatric Assessments and interventions

Domain	Assessment	Interventions
Cognition	Mini-Cog +/− MOCA<br>Collateral	TFT; B12; Folate; HbA1C<br>Memory Clinic
Mood	Geriatric Depression Scale	Cancer Support House<br>General Practitioner<br>Liaison Psychiatry<br>Psychiatry of Old Age
Functional Status	Katz ADL<br>Lawton IADL	Physiotherapy<br>Occupational therapy
Mobility	Falls<br>Timed Up and Go	Physiotherapy<br>Occupational therapy
Social Support	Medical Outcomes Study (MOS) Social Support Survey	Medical social worker<br>Cancer Support House<br>Irish Cancer Society<br>Social Prescribing
Nutrition	Mini Nutritional Assessment<br>Body mass index	Dietician
Polypharmacy	History<br>General Practitioner<br>Community Pharmacy	Pharmacist assessment:<br>Full drug reconciliation<br>Interaction check<br>Deprescribing

Abbreviations: MOCA: Montreal Cognitive Assessment; (I)ADL: (instrumental) activities of daily living.

Supplementary Table S2. Toxicity subgroups and CGA deficits.

Toxicities / CGA Variables	Subsequent Dose Reduction (*n* = 26) *n* (%)	Dose Delay (*n* = 52) *n* (%)	Discontinuation Due to Toxicity (*n* = 39) *n* (%)	Hospitalisation Due to Toxicity (*n* = 34) *n* (%)
Timed Up and Go				
Mean (seconds)	11.15	12.35	11.76	12.88
Median (range)	11 (8–17.1)	11.09 (8–31.6)	11.43 (8–18.06)	11.54 (8–31.6)
Falls in the last 6 months				
Yes	4 (15)	5 (10)	4 (10)	5 (15)
No	22 (85)	47 (90)	35 (90)	29 (85)
Concomitant meds				
Mean	6.14	5.96	5.89	5.9
Median (range)	6 (1–14)	6 (0–16)	6 (1–11)	6 (1–13)
Mini Nutritional Assessment				
0–7	9 (35)	17 (33)	13 (33)	12 (35)
8–11	10 (38)	26 (50)	18 (46)	17 (50)
12–14	7 (27)	9 (17)	8 (21)	5 (15)
Body mass index				
Mean	27.29	26.89	25.54	25.04
Median (range)	26.72 (15.43–36.16)	25.97 (15.43–39.25)	25.66 (15.43–39.25)	25.21 (15.43–34.14)
Geriatric depression scale				
≤5	21 (81)	43(83)	33 (85)	28 (82)
>5	5 (19)	8 (15)	4 (10)	4 (12)
N/A	0 (0)	1 (2)	2 (5)	2 (6)
Katz ADLs				
Mean	5.88	5.78	5.79	5.76
Median (range)	6 (5–6)	6 (3–6)	6 (4–6)	6 (4–6)
Lawton IADLs				
Mean	7.52	7.51	7.5	7.39
Median (range)	8 (5–8)	8 (3–8)	8 (4–8)	8 (4–8)
Charlson Comorbidity Index				
0	11(42)	24 (46)	15 (38)	13 (38)
1–2	10(38)	19 (37)	16 (41)	14 (41)
3–4	2(8)	4 (7)	3 (8)	3 (9)
≥5	3(12)	5 (10)	5 (13)	4 (12)
* Cognitive impairment				
Yes	7(27)	15 (29)	12 (31)	10 (29)
No	17(65)	31 (60)	22 (56)	20 (58)
N/A	2(8)	6 (11)	5 (13)	4 (12)
CARG toxicity risk				
Low	2(8)	2 (4)	1 (3)	1 (3)
Medium	8(30)	23 (44)	17 (43)	13 (38)
High	15(58)	24 (46)	20 (51)	19 (56)
N/A	1(4)	3 (6)	1 (3)	1 (3)

N/A = not available; Activities of Daily Living; Instrumental Activities of Daily Living; Cancer and Aging Research Group. *cognitive impairment defineds as MOCA <26
